# Whole-exome sequencing of DNA from peripheral blood mononuclear cells (PBMC) and EBV-transformed lymphocytes from the same donor

**DOI:** 10.1186/1471-2164-12-464

**Published:** 2011-09-26

**Authors:** Eric R Londin, Margaret A Keller, Michael R D'Andrea, Kathleen Delgrosso, Adam Ertel, Saul Surrey, Paolo Fortina

**Affiliations:** 1 Computational Medicine Center, Thomas Jefferson University Jefferson Medical College, Philadelphia, PA, USA; 2 National Molecular Laboratory, American Red Cross, Philadelphia, PA, USA; 3 The Coriell Institute for Medical Research, Camden, NJ, USA; 4 Cancer Genomics Laboratory, Kimmel Cancer Center; 5 Department of Cancer Biology, Cardeza Foundation for Hematological Research, Thomas Jefferson University Jefferson Medical College, Philadelphia PA, USA; 6 Department of Medicine, Cardeza Foundation for Hematological Research, Thomas Jefferson University Jefferson Medical College, Philadelphia PA, USA; 7 Department of Molecular Medicine, Universita' La Sapienza School of Medicine, Rome, Italy

## Abstract

**Background:**

The creation of lymphoblastoid cell lines (LCLs) through Epstein-Barr virus (EBV) transformation of B-lymphocytes can result in a valuable biomaterial for cell biology research and a renewable source of DNA. While LCLs have been used extensively in cellular and genetic studies, the process of cell transformation and expansion during culturing may introduce genomic changes that may impact their use and the interpretation of subsequent genetic findings.

**Results:**

We performed whole exome sequencing on a tetrad family using DNA derived from peripheral blood mononuclear cells (PBMCs) and LCLs from each individual. We generated over 4.7 GB of mappable sequence to a 125X read coverage per sample. An average of 19,354 genetic variants were identified. Comparison of the two DNA sources from each individual showed an average concordance rate of 95.69%. By lowering the variant calling parameters, the concordance rate between the paired samples increased to 99.82%. Sanger sequencing of a subset of the remaining discordant variants did confirm the presence of *de novo *mutations arising in LCLs.

**Conclusions:**

By varying software stringency parameters, we identified 99% concordance between DNA sequences derived from the two different sources from the same donors. These results suggest that LCLs are an appropriate representation of the genetic material of the donor and suggest that EBV transformation can result in low-level generation of *de novo *mutations. Therefore, use of PBMC or early passage EBV-transformed cells is recommended. These findings have broad-reaching implications, as there are thousands of LCLs in public biorepositories and individual laboratories.

## Background

One of the goals of genetic studies is to characterize genetic variation in individuals with specific conditions in order to identify variants associated with disease or efficacy of treatment modalities. Recently, massively parallel sequencing technology has made it possible for an individual's genome to be examined in fine detail. The increased use of this technology, often called next-generation (NGS) or deep sequencing, paired with powerful bioinformatic analyses of the resulting data, has facilitated the identification of novel disease-causing variants. Targeted sequencing of the genome's coding regions has been used to identify genes associated with rare monogenic disease including Kabuki syndrome [1], familial amyotrophic lateral sclerosis (ALS) [2], Miller syndrome [3] and Van Den-Ende-Gupta syndrome [4]. Currently, large sequencing projects, such as the 1000 Genomes project (http://www.1000genomes.org/) [5], are using this technology to characterize human genome variation on a population-based scale. As the cost of deep sequencing continues to decrease, the use of NGS technology will surely increase.

As deep sequencing projects are completed, additional DNA from study participants will be needed for replication and follow-up studies. While DNA derived from a subject's peripheral whole blood is a preferred source of starting genetic material, continued access to the participant for additional venipuncture may not be possible, or DNA isolated from peripheral whole blood may be available in limited quantities. Given these limitations, lymphoblastoid cell lines (LCLs) provide a convenient alternative. LCLs, created through the *in vitro *infection of B-lymphocytes with the Epstein-Barr virus (EBV), can provide an unlimited and lasting resource of the patient's genetic material. LCLs are well suited for many types of studies including genome-wide association [6,7], functional genomics [8], proteomics [9] and pharmacogenomics [10,11]. Furthermore, LCLs and their DNA can be made available to many investigators worldwide through biorepositories [12,13].

Despite the frequent use of LCL for biological research, concerns have been raised regarding potential genomic changes that may be introduced during cellular transformation and subsequent cell culturing. Several investigations have addressed this issue. For example, DNA copy number changes have been detected following extensive passaging of cell cultures [14]. The fidelity of genotype calls between DNA derived from LCLs and PBMCs from the same individual also has been examined [15-17]. These studies used gene chips to compare genotypes between the paired samples. Even though no significant changes were observed, this approach only interrogated the SNPs represented on the chips. Newly induced mutations may be introduced during the creation of the LCLs and/or after subsequent expansion of the derived cell lines. Recent studies have highlighted the association of *de novo *mutations with common disorders such as autism [18], schizophrenia [19] and mental retardation [20]. Therefore, determining if these mutations are real or an artifact of the starting material is of great importance as false-positive results can be introduced into the study design.

Recently, within the 1000 Genomes Project, the presence of *de novo *mutations in two trio families was described. The authors estimated that 0.61% of coding variants identified were *de novo *[5]. Since this study used DNA derived from LCLs, they were unable to compare the results to DNA derived from PBMCs in order to determine if these *de novo *mutations are real or induced through the cell transformation and culturing process.

The aim of the present study was to determine if DNA from EBV-transformed B-lymphocytes contains new mutations when compared to DNA from untransformed material. To address this, we performed whole exome-sequencing using both PBMC- and LCL-derived genomic DNA from a family of 4 individuals.

## Results and Discussion

**Figure 1:**
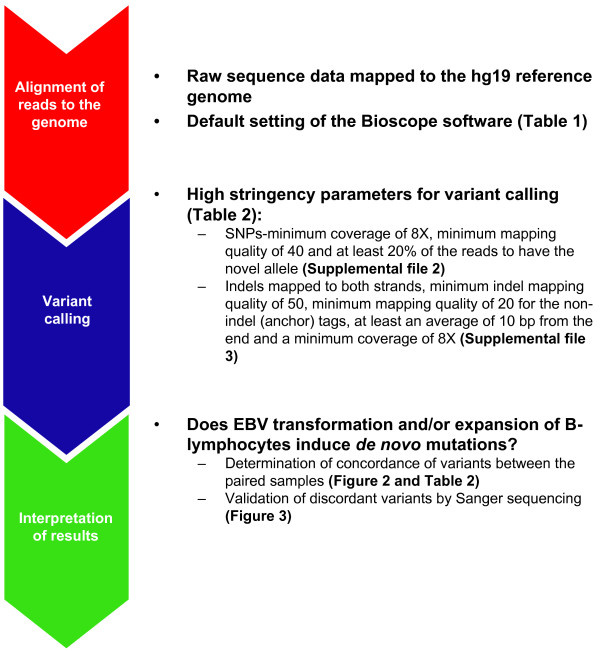
**Summary of the bioinformatic workflow followed**. The raw sequence data was aligned to the hg19 human genome build (UCSC). Following the alignment of the sequence data, high-stringency parameters were used to make SNP and indel calls. Following the identification of genetic variants, the interpretation of our results included comparing the two-paired samples sequenced to determine if *de novo *mutations arise following EBV-transformation of B-lymphocytes.

We performed whole exome sequencing on a tetrad family consisting of parents and two siblings, where DNA was derived from two sources, PBMCs and LCLs. Targeted capture efficiency and genomic variants were compared from the DNA derived from the two sources (Figure 1).

### Exome sequencing was performed to high-depth

We sequenced the exomes of 4 family members (Additional File 1) where the DNA from each individual was derived from two sources, PBMCs and LCLs. High stringency mapping parameters were used, which allowed for a maximum of 2 mismatches. The sequence was aligned to the hg19 genome build (UCSC). In total, an average of ~94 million sequence tags were generated per sample with 80.1% of the F3 tags and 67.1% of the F5 tags mapping to the genome (Table 1). This equated to an average of 5.97 GB of mappable sequence per sample. Removal of reads that mapped to multiple locations and outside of target regions resulted in an average of 80.4% of F3 tags and 79.4% of F5 tags uniquely mapped to the genome (Table 1). In total, an average of 4.77 GB of sequence was uniquely mapped to the genome. This represents an average read depth of 125X with 90.52% of the targeted sequence covered at least 8 times (Table 1). The targeted exomic capture was performed with similar efficiencies regardless of the initial DNA source (PBMC or LCL). A vast amount of robustly mapped sequence was generated. This suggests that targeted genomic capture and subsequent NGS can be performed successfully regardless of the source of the DNA.

### Exome sequencing identified high quality genomic variants

**Figure 2:**
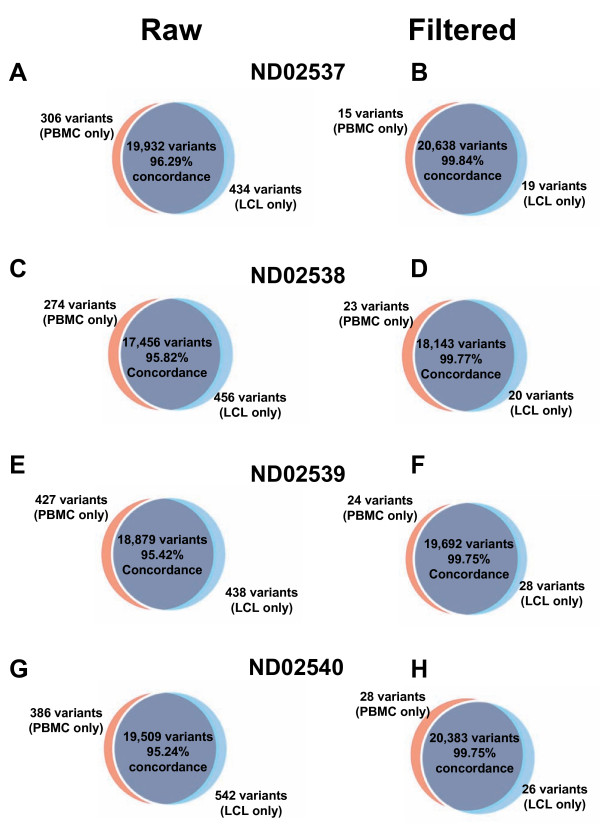
**Comparison of all variants identified from DNA derived from PBMCs and LCLs**. Venn diagrams showing the distribution of variants (SNPs and indels) identified for the DNA samples (**A, B**) ND02537, (**C, D**) ND02538, (**E, F**) ND02539 and, (**G, H**) ND02540. The dark blue represents the total variants that were in concordance between the paired samples. The light blue and red regions represent the total variants that were identified in only the LCL or PBMC sample respectively. **A, C, E **and **F **represent the initial concordance rates between the two paired samples, and **B, D, F **and **H **represent the concordance rates following the examination of discordantly identified variants.

The sequence data was used to identify and characterize genetic variants within the genome. High stringency SNP calling parameters were used (see Methods). This allowed us to identify variants with high confidence while reducing the possibility of false-positive results. In total, an average of 19,354 variants (SNPs and indels) were identified per sample (Table 2, Figure 2) with 7.39% being novel. Comparison between the paired samples, showed a 95.69% concordance rate.

In total, an average of 17,672 SNPs were identified per sample, with 2.75% of the SNPs being novel (Table 2 and Additional File 2). The majority of the identified SNPs (55.40%) were within the coding regions of the genome. These variants included synonymous SNPs and SNPs with potential functional impact on the gene's expression (Additional File 2). Additional SNPs located in known microRNAs were classified, although the functional effect of these SNPs is not known. The remaining SNPs (44.6%) were located within intronic or intergenic regions. These intergenic regions may represent pseudogenes, other non-characterized genes or additional regions coding for small non-coding RNAs.

An average of 1,682 indels was identified. Insertions ranging from 1-3 bp and deletions up to 11 bps were identified (Additional File 3). Of these, only a small percentage was within the coding region of the genome and the remaining variants were in intronic and intergenic regions. The finding that the number of indels in coding regions was small may be explained by the fact that the majority of these would result in frameshift mutations that would likely alter the protein product, including causing a premature stop.

### Fidelity of Variant Calls between different tissue sources

To examine the discordant calls, three steps were performed. First, the sequence quality of the discordant variants was examined to determine if the variant was not called in one of the two samples because it did not pass the variant-calling parameters. Second, discordant variants were examined in the context of the family pedigree. Finally, a subset of discordant SNPs was subjected to Sanger DNA sequencing analysis to confirm their presence.

Examining the variant-calling parameters (see Methods) revealed that the majority of discordant calls were due to the filtering parameters. Nearly 50% of these discordant variants were recovered by reducing from 20 to 15 percent the reads required to call a novel variant. Similarly, reducing the minimum read coverage from 8X to 5X recovered an additional 25% of the variants. Taken together, these results suggest that variant-calling parameters can be optimized to decrease discordant SNPs. By changing both the percentage of reads and read coverage settings, the concordance rate between sample types was increased from 96.33% to 99.82%.

**Figure 3:**
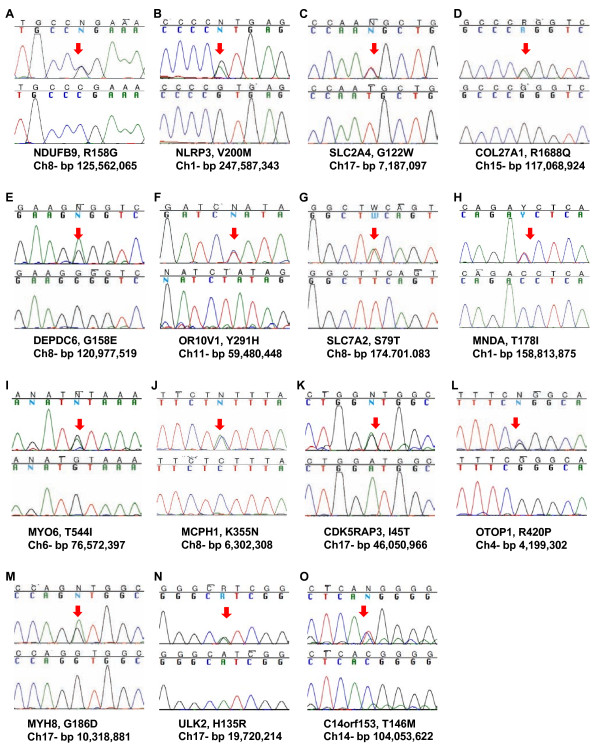
**Validation of identified variants, which were observed in only one of the DNA samples**. Each set of sequence chromatograms represents a different variant that was examined by Sanger sequencing. For each, the top panel is the sample with the SNP (highlighted with the red arrow), and the bottom is the corresponding sample with the non-variant allele. Below each chromatogram are the gene, putative amino acid change, and chromosome and bp location on the chromosome.

While the vast majority of variant discrepancies was due to sequencing artifacts and variant calling parameters, we did identify variants that were present in only one of the two-paired samples (Figure 2). These differences represented variants that were present in one of the two DNA samples (PBMC or LCL) for each subject. Together, a total of 183 variants were identified as being discordantly observed. Further examination of these SNPs revealed that 104 were both present in multiple samples, and represented within either the dbSNP or 1000 Genomes datasets, suggesting that they are not *de novo *variants. Interestingly, the remaining 79 variants were all observed in only a single LCL sample, and were not represented in dbSNP or 1000 Genomes (Additional File 4). Furthermore, we did identify a range of all 12 different types of nucleotide changes that can occur (Additional File 5) with C to T changes being the most common (~25%). Fifteen of these variants were selected for confirmation as being present in the LCL sample using Sanger sequencing, which confirmed their presence in the LCL samples only (Figure 3). Taken together, this result suggests that a small number of *de novo *mutations arose during the EBV-transformation process and/or subsequent culturing.

While we did identify variants that were present in the PBMC sample and not the LCL (Figure 2), several lines of evidence suggest that these are not *de novo *mutations. First, all of these variants were present within multiple family members, in particular parents and children. Second, recent studies of *de novo *mutations suggested that they were novel and not previously characterized; this was not the case here and in fact these variants have been characterized as being common (MAF >5%) based upon HapMap allele frequencies. While we would have expected to identify such variants within the PBMCs, the lack of *de novo *variants may be due to the targeting of only a small fraction of the genome for which no *de novo *mutations are present. Taken together, our results suggest an average concordance rate of 99.82% between the two-paired samples, leaving a *de novo *mutation rate of 0.18%. It should be noted that this *de novo *mutation rate is for the small portion of the genome sequenced and additional variants may be present in the rest of the genome.

## Conclusion

The aim of this study was to determine if lymphoblastoid cell lines were an acceptable source of DNA for deep sequencing studies. While previous studies have examined fidelity of known SNPs represented on gene chips [15-17], they did not explore the frequency of *de novo *mutations in LCL-derived DNA when compared to PBMC-derived DNA. We thoroughly examined this question using NGS technologies and performed whole-exome sequencing on a tetrad family where DNA was available from both PBMCs and LCLs. We explored the impact of variant-calling parameters and determined the effects of EBV transformation and culturing on the exome.

We did not observe significant differences in the overall coverage and the targeted exome capture efficiencies between samples derived from PBMCs or LCLs. Use of high stringency parameters resulted in an average 96.33% concordance rate of SNP calls between the two DNA sources (Table 2). Using lower stringency parameters resulted in a variant concordance rate of over 99%; and, furthermore, lowering the minimum mapping quality from 40 to 30 did not have a significant effect on the concordance rate. However, reducing the requirement of the number of novel allele counts from 20% to 15% had the greatest effect on increasing the variant concordance rate between the two DNA sources. Thus, the parameters used to identify variants in deep sequencing studies can greatly impact the results, and care should be taken with the interpretation of such results. Further examination of a subset of the remaining discrepant variants by Sanger sequencing confirmed (Figure 3) the presence of variants in one of the paired samples, suggesting up to a maximum of 1% of the discrepant variants may be the result of *de novo *mutation caused by EBV transformation and/or cell expansion.

We did observe a higher variability in the concordance rate among indels rather than SNPs. The short sequence reads produced make it challenging to identify indels from the sequence data. Since each sequence read is independently aligned to the reference genome (hg19), reads that may span an indel close to the ends of a read are difficult to align with gaps and can potentially be misaligned resulting in false SNPs. Furthermore, reads with indels may be aligned with multiple mismatches to the genome rather than a gap [21]. This greater difficulty in correctly mapping reads with indels to the reference genome may have resulted in the higher discordance rate observed between the two-paired samples.

*De novo *mutations have been identified as a cause for disorders such as autism [18], intellectual disability [20] and schizophrenia [19]. These studies highlight the importance of accurately identifying mutations when using exome sequencing. While we did not detect any *de novo *mutations in the siblings, we did identify differences between the two DNA sources. It is feasible that additional *de novo *mutations could be present within regions of the genome not covered in our sequencing. Our results are similar to those previously observed in the 1000 Genomes Project, which speculated that 0.61% of coding variants are *de novo*. In contrast to the 1000 genomes project, we were able to directly compare DNA derived from two sources from the same individual. While the number of *de novo *variants identified is a very small fraction of the total variants identified within the LCLs (an average of 0.18%), this rate represents only those within the targeted exome. Whole-genome sequencing experiments would no doubt identify additional variants. Thus, the presence of these variants may introduce false-positive findings into an experiment and further validation and replication of experimental results in additional samples would be required.

There are several limitations to this study. First, our approach focused on the exome, examining 1.22% of genome, and additional mutations may exist outside these regions. A more comprehensive approach, such as whole genome sequencing would allow for the interrogation of these regions. Second, LCLs from which the DNA was derived were in the pre-immortal state [9,22]. This represents the cell material commonly banked in biorepositories. In the pre-immortal stage, the EBV-transformed cells are actively proliferating and usually have normal diploid karyotypes without tumorigenic properties. After extensive population doublings (typically 160), LCLs reach a proliferative crisis in which pre-immortal cells die and post-immortal cells remain. These cells are often aneuploid and are able to differentiate indefinitely [9,22]. Thus, this study did not examine the effects that additional cell passaging may have on the genome. Third, it is possible that additional changes, such as copy number and loss-of-heterozygosity may be present in LCL-derived DNA; neither of which was examined here. This work suggests that LCLs are useful as a surrogate for DNA from PBMCs and an acceptable source material for disease association studies using exome sequencing. However, given the presence of a low level of *de novo *mutations occurring within LCLs, it is important to validate and confirm the results in independent sample sets to rule out the possibility of false-positive findings.

## Methods

### Subjects

A family of 4 individuals obtained from the NINDS Repository at the Coriell Institute for Medical Research (http://ccr.coriell.org/NINDS) was examined. This family (Family NINDS0254) consists of two parents (ND02538 and ND02539) and two siblings (ND02537 and ND02540). Subject ND02537 was diagnosed with idiopathic generalized epilepsy, with no seizures beginning at 3 years of age. All other members are unaffected. All subjects were collected under local IRB-approved protocols and submitted to the Repository as de-identified subjects.

### DNA extraction from peripheral blood

DNA was isolated by the Coriell Cell Repositories. DNA was isolated from 5-10 ml of peripheral blood mononuclear cells (PBMC) using the Gentra Puregene Blood Kit (Qiagen, Valencia, CA), according to the instructions of the manufacturer per Coriell Cell Repositories standard operating procedures (http://ccr.coriell.org/). Genomic DNA was examined for identity and relatedness using a set of 6 highly polymorphic microsatellites (THO, VWA31, D22s417, D5S592, D10S526, and FES/FPS).

### Establishment of and maintenance of lymphoblastoid cell lines (LCLs)

LCLs were established and maintained by Coriell Cell Repositories. Briefly, cell lines were established from freshly isolated lymphocytes using standard Epstein-Barr virus (EBV) transformation protocols that include mononuclear cell separation by gradient centrifugation and induced lymphocyte divisions by the mitogen Phytohemagglutinin (PHA). Briefly, peripheral blood was diluted with an equal volume of RPMI 1640 with 0.02 M HEPES, layered onto a Histopaque-1077 HybriMax (Sigma-Aldrich, St. Louis, MO) gradient, and centrifuged for 30 min at 400 X *g *at 18-20^0 ^C. The lymphocyte layer was harvested and washed twice in RPM1 1640 (Sigma-Aldrich, St. Louis, MO) with 0.02 M HEPES (Sigma-Aldrich, St. Louis, MO) and re-centrifuged. The resulting cell pellet was resuspended in 8 ml of cell culture medium and transferred to a 25-cm^2 ^cell culture flask containing 1 ml of EBV (prepared at Coriell Institute for Medical Research from a transformed marmoset cell line B95-8) and 1 ml of PHA reagent (Sigma-Aldrich, St. Louis, MO). The cells were incubated at 37^0 ^C in 5% (v/v) carbon dioxide, with medium changes twice each week. When a 4 x 10^6 ^total viable cell count was reached, the flask was subcultured and further expansions were obtained (1 x 10^8 ^total viable cells. All cell lines were established and grown in the absence of antibiotics. After transformation, the cells were cryopreserved to store the initial transformation. Resurrection of subsequent cultures starts with a cryopreserved primary passage with culturing performed to expand the culture and/or generate cells for DNA isolation. DNA was extracted from 4 x 10^7 ^cells by using the Gentra Puregene Blood Kit (Qiagen, Valencia, CA).

### Library generation and SOLiD sequencing

Three μg of genomic DNA was used for whole exome capture using the AB/Life Technologies SOLiD optimized SureSelect Human All Exon Target Enrichment System (Agilent Technologies). This kit performs in-solution hybridization with RNA oligonucleotides, enabling the specific targeting of approximately 38 Mb of the human genome (1.22% of the genome) covering ~18,000 genes. Following hybridization, 500 picomoles of the enriched exome library were used for emulsion PCR, to produce single DNA molecules on glass beads, which are then deposited onto a glass slide. Sequencing was performed on the SOLiD 4 instrument (Life Technologies Foster City, CA). Paired-end sequencing was performed. In this method, two ends of the same DNA fragment are sequenced in opposing directions which spans an inserted sequence of ~180 bp [23]. The two sequenced fragments are 50 bp (F3 tag) and 35 bp (F5 tag). When mapped back to the genome, the two-paired sequences should map to the same region and separated by a distance of the inserted fragment.

### Bioinformatics Pipeline

The AB SOLiD bioscope v1.3 software (Life Technologies, Foster City, CA) was used for data analysis, following three distinct steps (Figure 1). First, the color space reads were mapped to the hg19 reference genome (http://genome.ucsc.edu/) using an iterative mapping approach. Sequence coverage was determined as the proportion of targeted regions that was covered by at least one uniquely aligned read. Bases that aligned to the genome, but not in targeted regions were not considered for further analysis. Additionally, only regions that had greater than 8X coverage were considered for further analysis.

The second step of the bioinformatic pipeline was to identify genomic variants including SNPs and small insertion deletion variants (indels). SNPs were identified using the diBayes algorithm [24]. To determine the efficiency of variant calling, two separate stringency parameters were used. The first setting (high stringency) required variant calls on each strand with each base having a minimum coverage of 8X, a minimum base quality of 40, a strand minimum mapping quality of 40, and at least 20% of the reads to have the novel allele. Indels were detected using the SOLiD Small Indel Tool. Parameters for the identification of indels included that they be mapped to both strands, had a minimum indel mapping quality of 50 and minimum mapping quality of 20 for the non-indel (anchor) tags, at least an average of 10 bp from the end read position, and a minimum coverage of 8X.

SNPs and indels were annotated based upon their location within the hg19 reference genome. Variants were considered novel if they were not represented in either dbSNP build 132 and/or the 1000 Genomes project [5]. Variants were further characterized as being non-coding (intronic or intergenic) or coding (within an exonic region). These variants were characterized based upon their location or putative effect on the encoded protein: synonymous, non-synonymous, nonsense, splice-site, 5' or 3' UTR. Variants that were located within 50 bp from the start of a gene ("near gene") or in a microRNA also were characterized. Indels were characterized based upon the length of the inserted or deleted sequence, whether it was intronic, intergenic or exonic. The putative effect of the indel on the protein product was characterized using SeattleSeq Annotation (http://snp.gs.washington.edu/SeattleSeqAnnotation131/index.jsp) and examined for frameshift and changes in protein sequence.

The final step of the analysis included interpretation of the sequence results. Interpretation involved comparison of the identified variants between the DNA from both sources for each sample. The identified variants were compared to determine the (concordance rate or percentage of variants that were in common between the two DNA sources. Variants that were determined to be discordant were further examined to determine the source of discrepancies. Additionally, family inheritance patterns of the identified variants were examined.

### Variant validation

Variants for validation were chosen based upon three criteria: 1) being present in an LCL but not the corresponding PBMC sample; 2) the variant being observed in DNA from a child without being observed in either parent (*i.e.*, not displaying a family inheritance pattern); and, 3) being present within a coding region and predicted to alter the protein product (non-synonymous, splice site or nonsense mutations). A total of 15 variants were chosen forward for validation. The validation was performed using standard Sanger sequencing methods and analyzed on an AB 3730 DNA Analyzer (Applied Biosystems). PCR primers (Additional File 6) were designed to flank the regions under question and sequencing was performed from both strands.

## List of Abbreviations

LCL: lymphoblastoid cell line; EBV: Epstein-Barr virus; PBMC: peripheral blood mononuclear cell; SNP: single nucleotide polymorphism; indels: insertion-deletion mutations; NGS: next-generation sequencing.

## Competing interests

The authors declare that they have no competing interests.

## Authors' contributions

ERL, MAK, PF and SS conceived and designed the research study design. ERL and KD conducted experiments; ERL and AE analyzed the data; ERL, MAK, MRD and PF drafted the manuscript and all authors read and approved the final manuscript.

## Supplementary Material

Additional file 1 **Family NINDS02540**. Pedigree of family NINDS0254 used for exome sequencing. Click here for file

Additional file 2 **SNPs identified through exome sequencing**. Table listing the SNPs identified through exome sequencing in the four family members. Click here for file

Additional file 3 **Summary of insertion-deletion variants identified**. Table listing insertions-deletions identified through exome sequencing in the four family members. Click here for file

Additional file 4 **SNPs identified to be *de novo *in LCL samples**. Table listing the 79 variants identified as being *de novo*. Click here for file

Additional file 5 **Characterization of the types of nucleotide changes observed**. The table lists the type of observed nucleotide changes. Click here for file

Additional file 6 **SNPs selected for validation by Sanger sequencing**. Table listing the variants chosen for follow-up with primer sequences used for PCR and Sanger sequencing. Click here for file
