# Supplementary figures and images for: Whole-exome sequencing of DNA from peripheral blood mononuclear cells (PBMC) and EBV-transformed lymphocytes from the same donor
Source: BMC Genomics. 2011 Sep 26;12:464. doi: 10.1186/1471-2164-12-464 (PMC3203102; doi:10.1186/1471-2164-12-464)

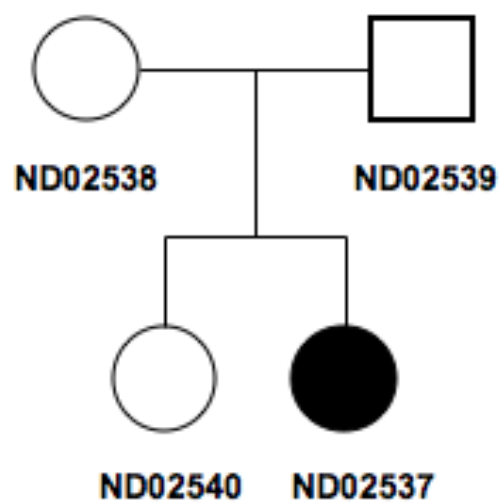

Supplement: Additional file 1 — Family NINDS02540. Pedigree of family NINDS0254 used for exome sequencing. [file 1471-2164-12-464-S1.PDF]
