# Supplementary material for: Whole-exome sequencing of DNA from peripheral blood mononuclear cells (PBMC) and EBV-transformed lymphocytes from the same donor
Source: BMC Genomics. 2011 Sep 26;12:464. doi: 10.1186/1471-2164-12-464 (PMC3203102; doi:10.1186/1471-2164-12-464)
Supplement: Additional file 2 — SNPs identified through exome sequencing. Table listing the SNPs identified through exome sequencing in the four family members. [file 1471-2164-12-464-S2.PDF]

Additional File 2. SNPs identified through exome sequencing

	**ND02537 (proband)**		**ND02538 (mother)**		**ND02539 (Father)**		**ND02540 (sister)**	
**DNA source**	PBMC	LCL	PBMC	LCL	PBMC	LCL	PBMC	LCL
Total SNPs	18660	18794	16184	16316	17531	17597	18079	18216
(novel)	(485)	(491)	(388)	(401)	(467)	(471)	(617)	(574)
Shared SNPs	18433		15963		17214		17806	
Variants unique to sample	227	361	221	353	317	383	273	410
SNP concordance rate between DNA sources	96.81%		96.40%		95.93%		96.16%	
Total non-coding SNPs	8599	8725	7486	7558	7710	7746	7489	7694
(novel)	(145)	(150)	(175)	(190)	(201)	(170)	(293)	(267)
% of SNPs in non-coding regions	46.08%	46.42%	46.26%	46.51%	43.98%	44.02%	41.42%	42.24%
Shared SNPs	7486		6392		7508		7439	
SNPs unique to sample	113	239	94	166	202	238	50	255
Total cSNPs	10061	10069	8698	8758	9821	9851	10590	10522
(novel)	(340)	(341)	(213)	(211)	(266)	(301)	(324)	(307)
% of SNPs in coding regions	53.92%	53.58%	53.69%	53.68%	56.02%	55.98%	58.58%	57.76%
Shared SNPs	9947		8571		9706		10367	
Discordant SNPs	114	122	127	187	115	145	223	155
(novel)	(8)	(9)	(11)	(9)	(2)	(37)	(28)	(11)
**Concordant SNPs**								
Synonymous	5036		4184		4955		5786	
(novel)	(119)		(64)		(97)		(114)	
Non-synonymous	4220		3772		4076		4879	
(novel)	(193)		(115)		(150)		(165)	
MicroRNA	5		4		5		2	
(novel)					(1)			
Near Gene	143		154		168		168	
(novel)	(5)		(5)		(5)		(2)	
Nonsense	40		30		30		45	
(novel)	(6)		(5)		(4)		(3)	
Splice	7		3		7		8	
(novel)							(1)	
UTR	488		424		46		479	
(novel)	(9)		(13)		(7)		(11)	
**Discordant SNPs**								
Synonymous	61	60	69	135	35	76	87	42
(novel)	(3)	(5)	(5)	(4)	(2)	(13)		(2)
Non-synonymous	57	54	36	50	78	64	135	111
(novel)	(5)	(4)	(5)	(5)		(22)	(28)	(9)
MicroRNA					1	1	1	2
Near Gene	2		8	1				
Nonsense (novel)			(1)	1				
Splice								
UTR	2					4 (2)		

Novel SNP: not represented in either dbSNP or 1000 Genomes Project Data

Near Gene: SNP located within 50 bp 3'- or 5'- of a gene
