# Supplementary material for: Whole-exome sequencing of DNA from peripheral blood mononuclear cells (PBMC) and EBV-transformed lymphocytes from the same donor
Source: BMC Genomics. 2011 Sep 26;12:464. doi: 10.1186/1471-2164-12-464 (PMC3203102; doi:10.1186/1471-2164-12-464)
Supplement: Additional file 3 — Summary of insertion-deletion variants identified. Table listing insertions-deletions identified through exome sequencing in the four family members. [file 1471-2164-12-464-S3.PDF]

Additional File 3. Summary of insertion-deletion variants identified

Sample	ND02537		ND02538		ND02539		ND02540	
DNA source	PBMC	LCL	PBMC	LCL	PBMC	LCL	PBMC	LCL
Total Indels	1578 (869)	1572 (889)	1586 (834)	1596 (841)	1775 (945)	1720 (978)	1816 (955)	1835 (944)
Shared between 2-paired samples	1422		1493		1665		1703	
Discordant indels	156	150	93	103	110	55	113	132
Indel Concordance Rate	89.86%		89.55%		90.09%		85.61%	
Coding	22 (12)	24 (15)	22 (14)	23 (16)	25 (16)	15 (15)	30 (22)	29 (15)
Frameshift	61 (29)	73 (37)	63 (37)	63 (33)	78 (43)	66 (37)	70 (40)	76 (40)
Intergenic	171 (117)	177 (123)	160 (115)	180 (119)	178 (148)	195 (135)	214 (157)	214 (156)
Intron	1223 (659)	1203 (665)	1242 (613)	1233 (621)	368 (661)	1322 (722)	1381 (666)	1385 (670)
Near-gene	20 (10)	15 (5)	21 (15)	15 (10)	25 (17)	23 (14)	24 (14)	25 (15)
Splice	8 (4)	10 (6)	5 (4)	11 (4)	8 (3)	12 (4)	13 (7)	9 (6)
UTR	73 (38)	70 (38)	73 (36)	71 (38)	193 (57)	87 (51)	84 (49)	78 (42)

Coding: Indels mapping to coding regions; Frameshift: Indels resulting in frameshift mutations; Intergenic: Indels mapping to intergenic regions; Intron: Indels mapping to intronic regions; Near-gene: Indels mapping within 100 bp 5'- or 3'- of a gene; Splice: Indels at a intron/exon splice junction; UTR: Indels within a 5' or 3' UTR region
