# Supplementary material for: Whole-exome sequencing of DNA from peripheral blood mononuclear cells (PBMC) and EBV-transformed lymphocytes from the same donor
Source: BMC Genomics. 2011 Sep 26;12:464. doi: 10.1186/1471-2164-12-464 (PMC3203102; doi:10.1186/1471-2164-12-464)
Supplement: Additional file 4 — SNPs identified to be de novo in LCL samples. Table listing the 79 variants identified as being de novo. [file 1471-2164-12-464-S4.PDF]

Additional File 4. SNPs identified to be *de novo* in LCL samples

Gene	Reference	Novel	Chromosome	BP	aa change	Sample observed in
API5	T	C	11	43342405	F89S	ND02537_LCL
OR10V1	A	G	11	59480448	Y291H	ND02537_LCL
LOC100131539	C	T	11	71530767	W21*	ND02537_LCL
MYH8	C	T	17	10318881	G186D	ND02537_LCL
ULK2	T	C	17	19720214	H135R	ND02537_LCL
ZNF675	G	A	19	23836616	Synonymous	ND02537_LCL
ANKRD27	C	G	19	33122338	M393I	ND02537_LCL
ALMS1	A	G	2	73675690	Y678C	ND02537_LCL
SLC23A3	G	A	2	220026624	3”-UTR	ND02537_LCL
KLHL18	G	A	3	47385389	Synonymous	ND02537_LCL
UGT2A3	C	T	4	69795626	A497T	ND02537_LCL
SLC9A3	G	A	5	482786	Synonymous	ND02537_LCL
POU5F1	G	A	6	31138107	Synonymous	ND02537_LCL
NCF1	C	T	7	74193709	Synonymous	ND02537_LCL
C9orf47	C	T	9	91605981	S24L	ND02537_LCL
SSX1	T	G	X	48121222	F101C	ND02537_LCL
MNDA	C	T	1	158813875	T178I	ND02538_LCL
NLRP3	G	A	1	247587343	V200M	ND02538_LCL
BBS2	G	A	16	56540107	Synonymous	ND02538_LCL
NLRP1	C	A	17	5462417	Q533H	ND02538_LCL
SLC2A4	G	T	17	7187097	G122W	ND02538_LCL
CAPS	G	A	19	5914470	R18H	ND02538_LCL
MARCH2	C	G	19	8491549	S78C	ND02538_LCL
ZNF331	C	T	19	54074878	Synonymous	ND02538_LCL
MAP3K13	G	A	3	185169151	A416T	ND02538_LCL
UGT2B17	G	A	4	69434217	5'-UTR	ND02538_LCL
PPAP2A	C	T	5	54771166	Synonymous	ND02538_LCL
DSP	A	G	6	7575637	Y849S	ND02538_LCL
FAM135A	A	T	6	71235353	K660*	ND02538_LCL
MCPH1	G	T	8	6302308	K355N	ND02538_LCL
NDUFB9	C	G	8	125562065	R158G	ND02538_LCL
NFASC	G	T	1	204978544	Intron	ND02539_LCL
ABCB10	A	T	1	229654670	Intron	ND02539_LCL

GALNT2	G	C	1	230390812	Intron	ND02539_LCL
LYST	G	C	1	235902048	Intron	ND02539_LCL
STAT2	A	C	12	56739986	Synonymous	ND02539_LCL
STK24	G	A	13	99109460	Synonymous	ND02539_LCL
OR11H4	A	G	14	20710968	Synonymous	ND02539_LCL
ADAMTSL3	G	A	15	84651866	Synonymous	ND02539_LCL
HYDIN	A	G	16	70993566	Synonymous	ND02539_LCL
HOOK2	C	T	19	12874383	E655K	ND02539_LCL
PSG2	C	G	19	43585111	G118	ND02539_LCL
VN1R4	G	A	19	53770764	R158G	ND02539_LCL
TPO	G	C	2	1499841	C696S	ND02539_LCL
EHBP1	A	G	2	63176179	Y733C	ND02539_LCL
SCTR	C	T	2	120209633	A292T	ND02539_LCL
TTN	T	A	2	179644024	N1253Y	ND02539_LCL
SSFA2	A	T	2	182786966	T1168S	ND02539_LCL
COL4A3	C	T	2	228176554	R1661C	ND02539_LCL
OTOP1	C	G	4	4199302	R420P	ND02539_LCL
MYO6	C	T	6	76572397	T544I	ND02539_LCL
BCLAF1	C	A	6	136599885	R43M	ND02539_LCL
MCPH1	G	T	8	6302308	K355N	ND02539_LCL
SLC7A2	T	A	8	17401083	S79T	ND02539_LCL
IGSF3	C	A	1	117142700	S631I	ND02540_LCL
NFRKB	C	T	11	129735822	Synonymous	ND02540_LCL
ZNF84	A	C	12	133634450	Synonymous	ND02540_LCL
USP12	T	C	13	27669852	Synonymous	ND02540_LCL
NAA16	C	T	13	41894867	Synonymous	ND02540_LCL
C14orf153	A	T	14	104053622	T146M	ND02540_LCL
HERC2	G	A	15	28475625	T1566M	ND02540_LCL
ACSM3	C	T	16	20792417	H302Y	ND02540_LCL
HYDIN	G	T	16	71015329	P1491H	ND02540_LCL
IMP5	G	A	17	43923180	R303H	ND02540_LCL
KIAA1267	G	C	17	44248499	Synonymous	ND02540_LCL
CDK5RAP3	T	C	17	46050966	I45T	ND02540_LCL
TEX2	G	A	17	62290699	Synonymous	ND02540_LCL
LRG1	G	A	19	4538780	Synonymous	ND02540_LCL

NA	G	C	21	15135926	intergenic	ND02540_LCL
ATF4	A	G	22	39918175	I208M	ND02540_LCL
FAM116A	C	T	3	57631400	R342H	ND02540_LCL
ARSJ	T	C	4	114824366	I288M	ND02540_LCL
BAT2	G	A	6	31602967	R1740H	ND02540_LCL
HSPA1A	G	C	6	31783507	5'-UTR	ND02540_LCL
CHN2	A	T	7	29252724	Intron	ND02540_LCL
URGCP	G	T	7	43959518	Intron	ND02540_LCL
SFTPC	G	A	8	22020201	A53T	ND02540_LCL
DEPDC6	G	A	8	120977519	G158E	ND02540_LCL
COL27A1	G	A	9	117068924	R1688Q	ND02540_LCL
