# Supplementary material for: Whole-exome sequencing of DNA from peripheral blood mononuclear cells (PBMC) and EBV-transformed lymphocytes from the same donor
Source: BMC Genomics. 2011 Sep 26;12:464. doi: 10.1186/1471-2164-12-464 (PMC3203102; doi:10.1186/1471-2164-12-464)
Supplement: Additional file 5 — Characterization of the types of nucleotide changes observed. The table lists the type of observed nucleotide changes. [file 1471-2164-12-464-S5.PDF]

Additional File 5. Characterization of the types of nucleotide changes observed

Type of Change	Number
A to C	2
A to G	7
A to T	5
C to A	3
C to G	5
C to T	16
G to A	21
G to C	6
G to T	6
T to A	2
T to C	5
T to G	1
