# Supplementary material for: Whole-exome sequencing of DNA from peripheral blood mononuclear cells (PBMC) and EBV-transformed lymphocytes from the same donor
Source: BMC Genomics. 2011 Sep 26;12:464. doi: 10.1186/1471-2164-12-464 (PMC3203102; doi:10.1186/1471-2164-12-464)
Supplement: Additional file 6 — SNPs selected for validation by Sanger sequencing. Table listing the variants chosen for follow-up with primer sequences used for PCR and Sanger sequencing. [file 1471-2164-12-464-S6.PDF]

Additional File 6. SNPs selected for validation by Sanger sequencing

Gene	Reference allele	Novel allele	Chromosome	Coordinate	AA change	Sample code	Forward Primer	Reverse Primer
OR10V1	A	G	11	59480448	Y291H	ND02537_LCL	TTAGCATCCCCCTCTCATTG	CTCCCAAAGTGCTGGGATTA
MYH8	C	T	17	10318881	G186D	ND02537_LCL	CGAGAGAATCAGTCCATCCTG	GGCGCTGATGATTTGATCTT
ULK2	T	C	17	19720214	H135R	ND02537_LCL	AATATTCGGGAGGCTGAGGT	AAGGACTGACCCCTGGAGTT
NDUFB9	C	G	8	125562065	R158G	ND02538_LCL	TTCCTTCCTTGCCTCCTTCT	ACAGTGGCACAGTCACTTGG
MNDA	C	T	1	158813875	T178I	ND02538_LCL	GTTTCCAAGGACATCCCATC	CATGTGGCTTCTCACCAATG
NLRP3	G	A	1	247587343	V200M	ND02538_LCL	GCCTCAACAAACGCTACACA	TGTGTCACAAGGCTCACCTC
SLC2A4	G	T	17	7187097	G122W	ND02538_LCL	TCTCAGTGGCTTGGAAGGTT	CAGGTGAGTGGGAGCAATCT
C14orf153	A	T	14	104053622	T146M	ND02540_LCL	CCTTTCAGGTCAGAAAGCAA	AGCTTGCAGTGAGCCAAGAT
MYO6	C	T	6	76572397	T544I	ND02539_LCL	TTGAAGCCAAATTAGTGGGAAT	CCCACCTCAGCTTCCCTAGT
SLC7A2	T	A	8	17401083	S79T	ND02539_LCL	GATCCGGAGAAAAATCGTGA	ATTCCAGCCAGTGATGAAGG
MCPH1	G	T	8	6302308	K355N	ND02539_LCL	TCTTCAACAAAAGGCCACCT	CAGTTTTCCGAGGACTGGAG
OTOP1	C	G	4	4199302	R420P	ND02539_LCL	ATCTCCTGGGGCTCAATCTT	GACTTTTCTCTTGGCGTTGC
CDK5RAP3	T	C	17	46050966	I45T	ND02540_LCL	CCCATGACCCCCATACTTTA	GCCCTGGTGGCTAAATAACA
COL27A1	G	A	9	117068924	R1688Q	ND02540_LCL	AGCCTCTGAGCCATTGTGAT	GGCGGAAATCAAATACTCCA
DEPDC6	G	A	8	120977519	G158E	ND02540_LCL	GTTGCAGTGAGCCAAGATCA	ATACGCTCACCATGCTGGAT

Reference allele: allele in hg19 reference genome; Novel allele: allele identified by exome sequencing; Coordinate: bp location on chromosome; AA change: amino acid change; Sample code: sample in which the mutation was detected; Forward/Reverse primer: Primer sequences used for Sanger sequencing
